# Art therapy interventions and developmental outcomes in children with ASD: a systematic review

**DOI:** 10.3389/fpsyg.2026.1771615

**Published:** 2026-09-23

**Authors:** Qiuxia Zhu, Fanxuan Kong

**Affiliations:** Faculty of Art, Lanzhou University of Finance and Economics, Lanzhou, Gansu, China

**Keywords:** art therapy, autism spectrum disorder, children with ASD, creative and nonverbal intervention, developmental outcomes

## Abstract

Although the prevalence of autism spectrum disorder (ASD) has increased globally, evidence-based interventions that address both emotional and social dimensions in low-cost, real-world settings remain limited. Art therapy, characterized by nonverbal and multisensory engagement, has been applied in clinical and educational contexts for children with ASD. However, recent studies have largely emphasized overall outcomes, with limited synthesis of intervention processes and research trends. This systematic review followed PRISMA 2020 guidelines. A search of Web of Science, Scopus, and PubMed identified 915 records, of which 22 empirical studies (2016–July 2025) were included, covering quantitative, qualitative, and mixed-method designs involving art therapy interventions for children with ASD. Study characteristics were extracted and synthesized using a narrative thematic synthesis approach. The findings identify four recurring themes: (1) Emotional Expression and Regulation, (2) Social Skills Development, (3) Creative and Nonverbal Processes, and (4) Sensory and Relational Integration. Across studies, interventions commonly involved multisensory art-making, nonverbal expression, and relational engagement within structured social contexts. Building on these four themes, the Creative–Relational Art Therapy Framework (CRATF) is proposed as a preliminary conceptual framework that integrates emotional, social, creative, and sensory–relational aspects, offering a basis for future intervention design and empirical evaluation in clinical and educational settings.

## 1 Introduction

### 1.1 Research background

In recent years, the reported prevalence of autism spectrum disorder (ASD) has increased (İnci and Saǧlam, 2025). Since the precise etiology of ASD remains unclear, no definitive cure is currently available, and targeted pharmacological interventions remain limited (Huili et al., 2023). Early intervention is therefore widely regarded as an important approach for supporting developmental outcomes and quality of life in children with ASD (Koo and Thomas, 2019).

Conventional interventions such as behavioral interventions and speech therapy have demonstrated effectiveness. However, they may involve high costs, limited availability of trained professionals, and prolonged training periods. These constraints can pose challenges to sustained implementation, especially in low- and middle-income nations and among families with limited financial resources (Huili et al., 2023). For this reason, art therapy has increasingly been considered as a promising supplementary approach because of its nonverbal and sensory-based forms of engagement, which may be more adaptable across different intervention settings.

Existing studies suggest that art therapy may support self-awareness, behavioral regulation, sensory integration, and emotional expression in children with ASD (Durrani, 2020). Although much of this research consists of case studies and small-scale samples, researchers have increasingly examined its potential contributions to social, emotional, and behavioral outcomes (Schweizer et al., 2019). Art therapy encompasses diverse forms such as painting, music, dance, and drama. These modalities offer nonverbal means of expression and communication, which may be particularly relevant for children with limited verbal abilities or difficulties in emotional regulation. Some researchers also note that many children with ASD are visual learners who process images more easily than verbal language. Art therapy may align with this strength by using visual media to support interpersonal connection, emotional expression, and social engagement (Koo and Thomas, 2019).

The Parent-Child Art Therapy model has also received increasing attention. This approach emphasizes the joint participation of the child and their primary caregiver, aiming to improve the quality of parent-child interactions and strengthen the family support system. Beyond supporting children's developmental progress, the model also highlights parental participation and reflection during the therapeutic process. Such involvement may contribute to the sustainability and transferability of intervention experiences across settings (Bitan and Regev, 2022). This perspective is relevant to the present review as it highlights the role of contextual and relational factors in shaping intervention processes and outcomes.

### 1.2 ASD

ASD is a neurodevelopmental disorder characterized by persistent deficits in social communication and interaction skills, accompanied by repetitive and restricted patterns of behavior and interests (Abdulah et al., 2024). According to the Diagnostic and Statistical Manual of Mental Disorders, Fifth Edition (DSM-5), symptoms typically emerge in early childhood and may affect multiple domains. These include language and speech development, sensory processing, and social cognition, as well as limited theory of mind, repetitive and stereotyped behaviors, and restricted interests (Durrani, 2021).

ASD presents along a spectrum of symptom severity. Some individuals display relatively mild characteristics with average intellectual functioning, while others may experience more substantial developmental challenges (Broupi et al., 2023). Notably, children with ASD exhibit high heterogeneity in language development. Previous research suggests that approximately 40% lack any spoken language, while the remainder may experience significant difficulties in language comprehension and use (Thayer and Bloomfield, 2021). Sensory integration dysfunction (SID) is another commonly reported comorbidity, which may present as either hypersensitivity or under-responsiveness to environmental stimuli, which can affect anxiety levels, behavioral regulation, and social interaction (Huili et al., 2023).

In summary, ASD is generally understood as a multidimensional neurodevelopmental disorder characterized by considerable individual variability. This diversity suggests the need for flexible and individualized approaches in intervention and support.

### 1.3 Art therapy

Art therapy is a psychotherapeutic approach that uses visual arts as a medium, delivered by trained therapists within a structured therapeutic relationship. It aims to support emotional expression, self-regulation, and relationship-building through nonverbal sensory and motor experiences (Broupi et al., 2023).

Children with ASD often experience challenges related to social communication, sensory integration difficulties, emotional regulation, and repetitive behaviors (Abdulah et al., 2024). Art therapy may offer a safe environment in which sensory intensity can be adjusted using diverse materials such as paints, clay, and foam. Allowing children to select their own materials can support sensory regulation and gradual desensitization, which may help reduce anxiety and enhance attention (Thayer and Bloomfield, 2021). The creative process also involves nonverbal interaction between the therapist and the child. Two key relational processes in this context are “tuning” (the therapist's attentive, moment-by-moment responsiveness to the child's emotional and sensory cues) and “mirroring” (the therapist's reflection of the child's body language, gestures, or artistic expressions to communicate understanding and connection). These interactions may help reconstruct attachment experiences at the relational level and support social reciprocity (Koo and Thomas, 2019). Some studies report that short-term, intensive art therapy interventions may improve communication, emotional regulation, and peer interaction among children with ASD, with effects that show some transferability across contexts (Lipska and Leś, 2023). In this sense, art therapy is often described as a multisensory and relationship-oriented approach that may support the social–emotional development in children with ASD, owing to its minimal verbal demands and focus on relational engagement.

### 1.4 Problem statement

Although many studies have examined the potential of art therapy for children with ASD, a systematic review that comprehensively maps study designs, intervention approaches, and outcomes across this literature remains limited. Previous reviews have primarily focused on overall efficacy, leaving limited discussion of the intervention mechanisms, methodological trends, and emerging practices in this field. To address these gaps, this study reviews literature published between 2016 and 2025, guided by two research questions:

RQ1: What methodological characteristics and research trends have emerged in art therapy studies for children with ASD over the past decade?
RQ2: How do art therapy interventions relate to developmental outcomes in children with ASD?

In addressing these questions, the review considers studies examining a range of art therapy modalities, including visual arts, music, dance, and multimodal practices, across qualitative, quantitative, and mixed-method designs. Relevant outcomes include emotional regulation, social communication, and sensory integration, as well as child-level characteristics of cognitive profiles and sensory preferences. Contextual elements such as parental involvement and therapist stance are also considered. In addition, the review notes emerging developments such as digital and online art-making platforms, which may broaden access to art-based interventions. The review aims to synthesize existing research and outline directions for future studies on art therapy interventions for children with ASD.

## 2 Methodology

### 2.1 Protocol registration

This systematic review followed the Preferred Reporting Items for Systematic Reviews and Meta-Analyses (PRISMA 2020) guidelines (Page et al., 2021). To enhance methodological transparency and reproducibility, the review protocol was registered in the International Platform of Registered Systematic Review and Meta-analysis Protocols (INPLASY; Registration ID: INPLASY2025110073). The protocol defined the research objectives, eligibility criteria, databases, search strategies, and quality appraisal procedures before data collection.

### 2.2 Search strategy and data sources

A systematic literature search was conducted in accordance with PRISMA 2020 guidelines across three databases: Web of Science (WOS), Scopus, and PubMed. The aim was to identify peer-reviewed empirical studies examining art therapy interventions for children and adolescents with ASD. Publications dated between 1 January 2016 and 1 July 2025 were included, with no restrictions on study location.

The search strategy combined controlled vocabulary, for example, MeSH terms in PubMed, with free-text keywords. Boolean operators AND and OR were applied to link concepts relating to art therapy, autism, and child or adolescent populations. A brief description of the search logic is presented below, while the full search strings and retrieval counts for each database are summarized in Table 1.

**Table 1:** Database search strategy and records retrieved.

Database	Search string applied	Records retrieved (*n*)
Web of science (WOS)	(“Art therapy”) AND (“Autism Spectrum Disorder” OR “ASD” OR “Autistic Disorder”) AND (“Children” OR “Adolescents” OR “Youths” OR “Pediatric Population” OR “Kids”).	n = 262
Scopus	(“Art therapy”) AND (“Autism Spectrum Disorder” OR “ASD” OR “Autistic Disorder”) AND (“Children” OR “Adolescents” OR “Youths” OR “Pediatric Population” OR “Kids”).	*n* = 524
PubMed	(“Art therapy”) AND (“Autism Spectrum Disorder” OR “ASD” OR “Autistic Disorder”) AND (“Children” OR “Adolescents” OR “Youths” OR “Pediatric Population” OR “Kids”).	*n* = 129

The searches were limited to English-language, peer-reviewed journal articles, which may introduce language bias and should be considered when interpreting the findings. Additional records were identified through manual screening of reference lists. All database searches were completed on July 1, 2025.

### 2.3 Eligibility criteria

Eligibility criteria were defined in advance following the PICOS framework (Population, Intervention, Comparison, Outcomes, and Study Type). These criteria aimed to identify empirical studies examining developmentally oriented art therapy interventions for children with ASD. Both inclusion and exclusion criteria are summarized in Table 2 and were applied during the title–abstract screening and full-text review stages.

**Table 2:** Eligibility criteria based on the PICOS framework.

Component	Inclusion criteria	Exclusion criteria
Population (P)	Children and adolescents aged 2–18 years diagnosed with autism spectrum disorder (ASD).	Studies involving adults, mixed samples without disaggregated data, or non-clinical populations.
Intervention (I)	Studies explicitly implementing art therapy or creative arts therapy as the primary or adjunctive intervention.	General art education, recreational art activities, digital art classes without therapeutic intent, or interventions unrelated to art therapy.
Comparison (C)	Both controlled and uncontrolled empirical designs were eligible.	Studies without empirical data (e.g., theoretical or conceptual papers).
Outcomes (O)	Reported developmental outcomes related to emotional regulation, social communication, sensory integration, creativity, or behavioral adaptation.	Studies lacking measurable outcomes or reporting academic/skill-based outcomes unrelated to developmental change.
Study type (S)	Peer-reviewed quantitative, qualitative, or mixed-methods empirical studies published in English between 2016 and 2025.	Reviews, meta-analyses, conference papers, book chapters, dissertations, gray literature, and non-English publications.

This framework guided the selection of studies that provide empirical evidence of art therapy interventions for children with ASD, with attention to measurable developmental outcomes while excluding purely descriptive or theoretical accounts.

### 2.4 Screening and selection process

A total of 915 records were identified through database searching (Web of Science, *n* = 262; Scopus, *n* = 524; and PubMed, *n* = 129). All retrieved records were imported into Rayyan reference management software. After removing 74 duplicate records, 841 unique records remained for screening.

The screening process followed the PRISMA 2020 recommendations and was conducted independently by two reviewers to reduce potential selection bias. Titles and abstracts were first screened to determine relevance to the review objectives. During this stage, 577 records were excluded for not meeting the inclusion criteria, most commonly because they were unrelated to art therapy, did not involve child participants, or were published outside the defined time range. A total of 264 full-text reports were sought for retrieval. Among these, 19 reports could not be retrieved. The remaining 245 reports were assessed for eligibility. After full-text review, 223 studies were excluded for the following reasons: (a) review or conceptual papers (*n* = 21); (b) insufficient focus on art therapy as the primary intervention (*n* = 202). Ultimately, 22 studies met all eligibility criteria and were included in the final synthesis.

Any discrepancies between reviewers were discussed until consensus was reached. The full screening procedure and exclusion reasons are presented in the PRISMA 2020 flow diagram (Figure 1), which illustrates each stage from identification to final inclusion.

**Figure 1:**
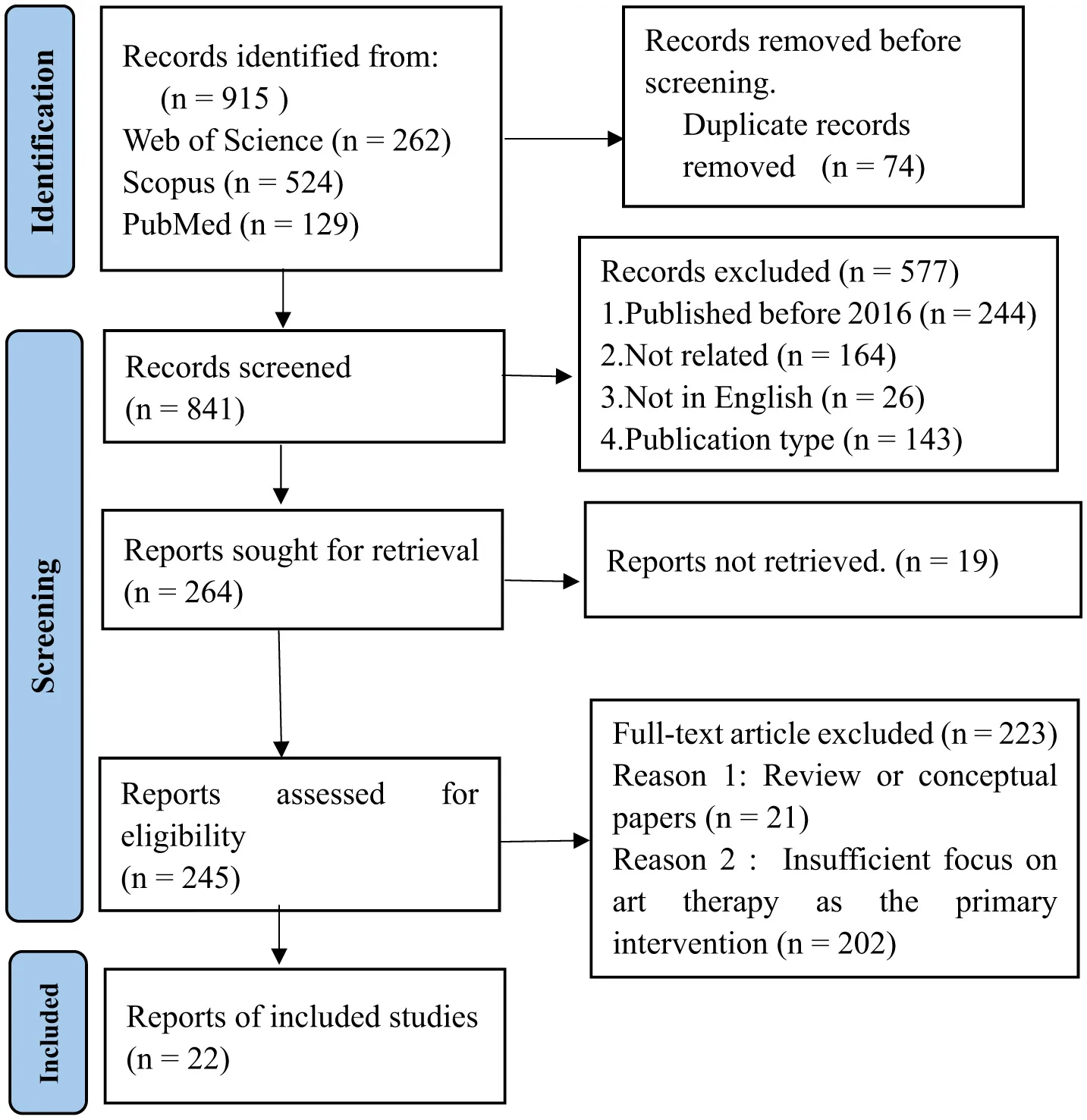
PRISMA flow diagram of the research process.

### 2.5 Data extraction

Key bibliographic and methodological information, such as author, year, country, participant age range, intervention type, duration, sample size, study design, and main outcomes, was extracted using a standardized data extraction form into an Excel matrix. Data extraction was conducted independently by two authors, and any discrepancies were resolved through discussion until consensus was reached.

As shown in Appendix A, the 22 included studies represented approximately 546 children and young people with ASD in total. Individual sample sizes ranged from 1 to 200 participants, indicating considerable variability in study designs and intervention contexts. Because some studies reported sample-size ranges, professional perspectives, or planned samples rather than exact numbers of directly enrolled child participants, this total should be interpreted as approximate.

### 2.6 Quality appraisal

All included studies were evaluated using the Crowe Critical Appraisal Tool (CCAT), which was selected due to its applicability to both qualitative and quantitative research designs. The CCAT assesses eight domains: preliminaries, introduction, design, sampling, data collection, ethical considerations, results, and discussion (Crowe et al., 2012). Each domain is scored from 0 to 5, yielding a maximum of 40 points.

Two reviewers independently rated all studies; inter-rater agreement exceeded 90%. The total CCAT scores ranged from 30 to 38 (mean = 34.4, SD = 2.21). A summary of individual study scores is provided in Appendix B.

### 2.7 Data synthesis

Due to the heterogeneity of study designs, outcome measures, and intervention modalities, a quantitative meta-analysis was not feasible. Instead, a narrative thematic synthesis was applied following Braun and Clarke's six-phase framework: (1) familiarization, (2) coding, (3) theme identification, (4) theme review, (5) theme definition, and (6) interpretation (Braun and Clarke, 2019).

Both descriptive quantitative and qualitative analytical procedures were employed. Quantitative analysis involved summarizing bibliographic and methodological characteristics of the included studies, such as publication year, geographical distribution, intervention types, and sample characteristics. The qualitative analysis was performed using ATLAS.ti 9 software to support systematic coding and theme development. This approach to thematic synthesis was informed by the procedures outlined by Zairul (2021), who employed ATLAS.ti for systematic literature-based thematic analysis. In the present study, this framework guided the coding structure and the iterative development of themes. All 22 studies were uploaded into ATLAS.ti 9 for inductive coding. Codes were assigned based on article metadata and substantive elements, including research objectives, design, and conclusions. High-frequency thematic keywords were identified and iteratively refined. Overlapping subthemes were consolidated through repeated review, with related subthemes grouped under higher-order themes to form a finalized thematic framework. The resulting thematic framework comprised four interrelated domains: Emotional Expression and Regulation, Social Skills Development, Creative and Nonverbal Intervention, and Sensory–Relational Integration.

This systematic procedure enhances the transparency and reproducibility of the review and contributes to methodological rigor.

## 3 Results

The results are presented in two sections, beginning with the study attributes of the included articles, followed by the thematic synthesis findings.

### 3.1 Article attributes

This section summarizes the structural characteristics of the included studies, including publication year, geographic distribution, thematic focus, and journal outlets.

Between 2016 and 2025, 22 empirical studies met the inclusion criteria (mean = 2.2 publications per year). Annual output ranged from one to four publications (Figure 2). Overall, publication output showed a gradual increase over the past decade, with minor fluctuations. The relatively low number of publications in 2025 reflects database indexing status at the time of data collection (search completed on 1 July 2025).

**Figure 2:**
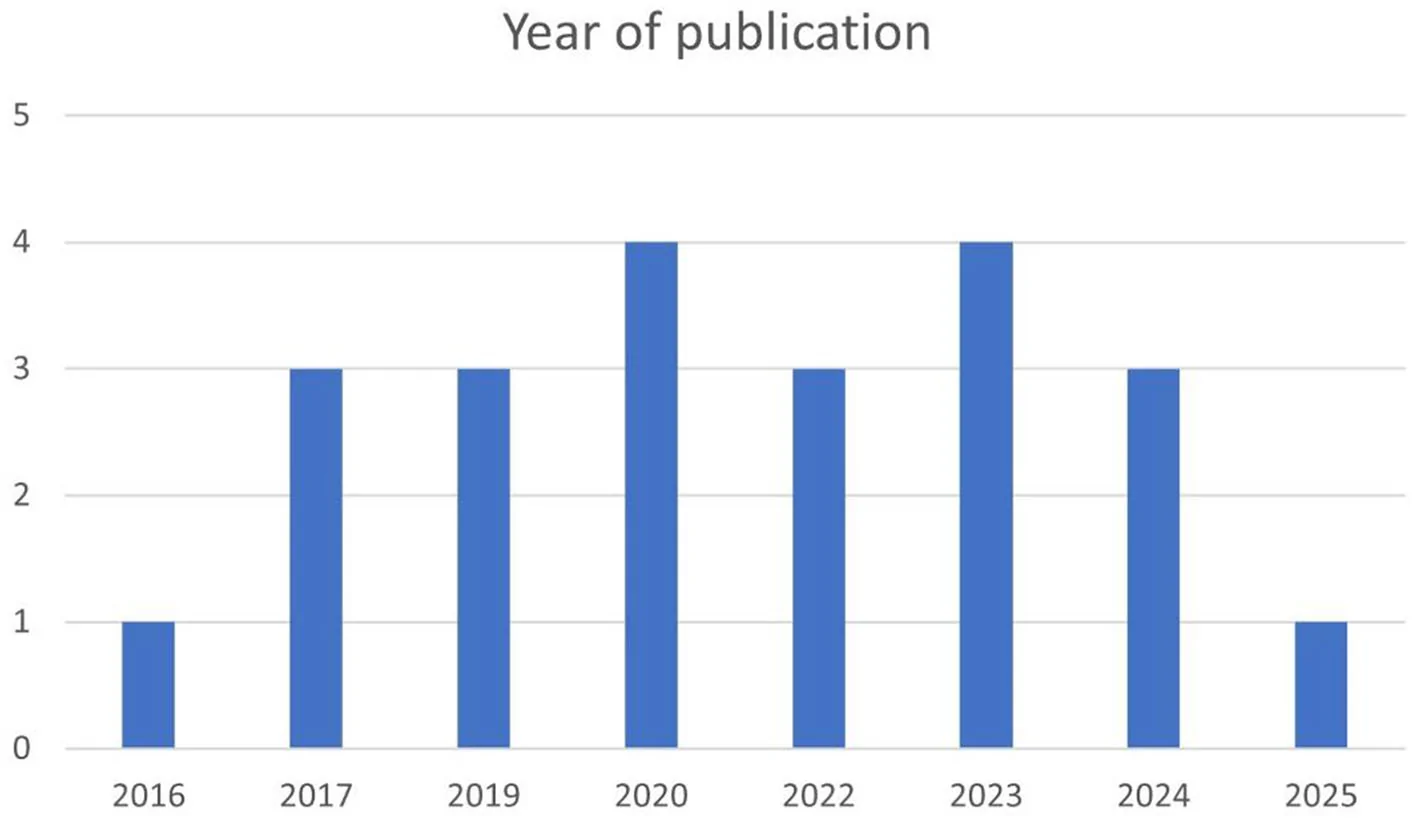
Year of publication.

In terms of geographic distribution, studies were conducted across Europe, North America, Asia, and Oceania (Figure 3). Europe and North America together accounted for 12 studies (54.5%), including four from the United States and four from the Netherlands, with additional contributions from the United Kingdom, Canada, Poland, and Greece. Studies from Asia and transcontinental or Middle Eastern contexts accounted for nine studies (40.9%), including two from China and one each from Singapore, India, Indonesia, Iraq, Israel, Saudi Arabia, and Turkey. One study was conducted in Australia. The distribution indicates that research activity was primarily located in Europe and North America, with additional contributions from Asian and Middle Eastern contexts.

**Figure 3:**
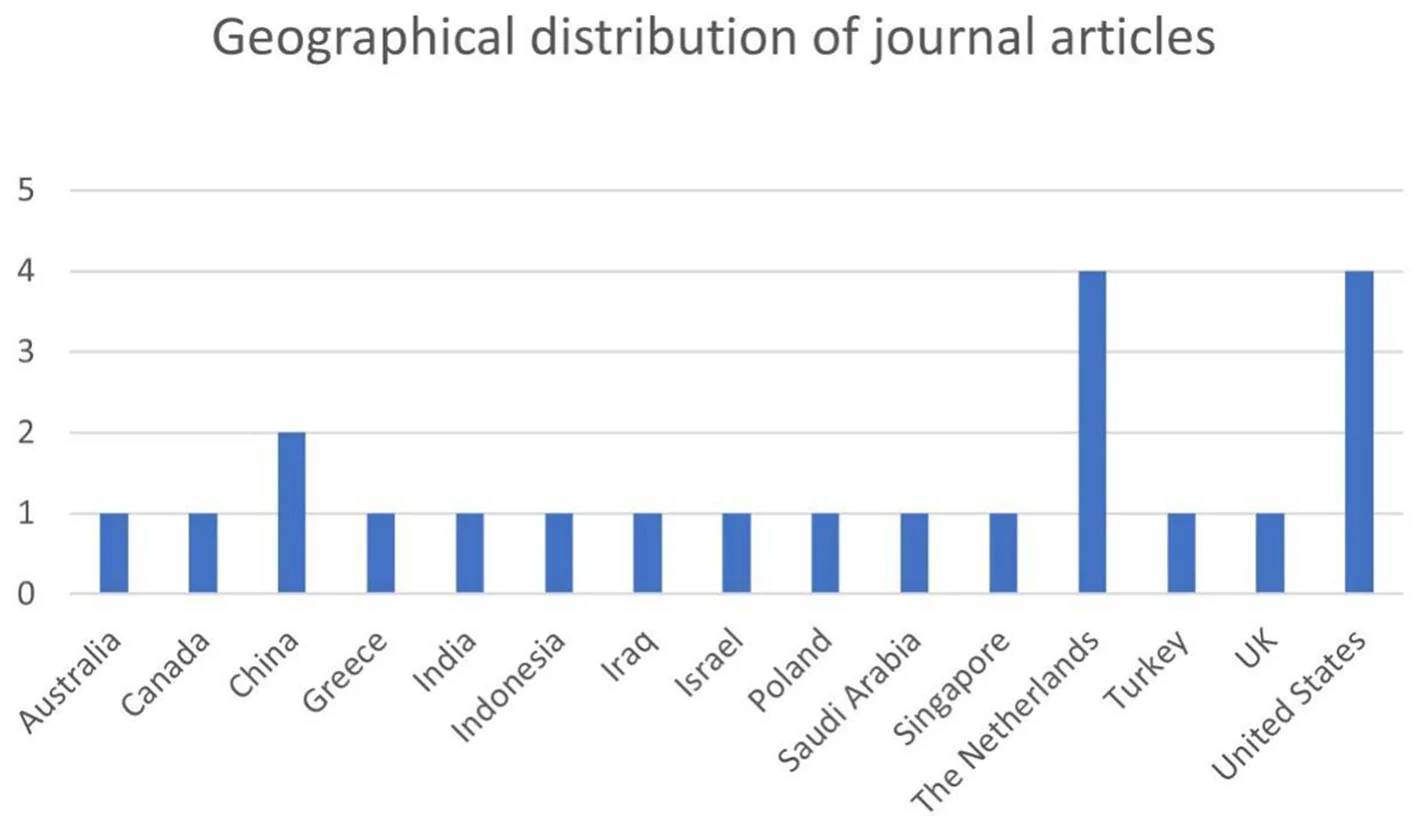
Geographical distribution of journal articles.

Table 3 presents the distribution of studies across thematic domains. Social Skills Development appeared most frequently among the included studies. Emotional Expression and Regulation and Sensory–Relational Integration were also represented across multiple studies. Creative and Nonverbal Intervention appeared in comparatively fewer publications.

**Table 3:** Distribution of included studies across thematic domains.

Authors & Year	Creative and nonverbal intervention	Emotional expression and regulation	Sensory and relational integration	Social skills development
Abdulah et al. (2023)				**√**
Abdulah et al. (2024)				**√**
Bitan and Regev (2022)			**√**	
Broupi et al. (2023)				**√**
Chapman and Evans (2020)	**√**	**√**	**√**	
Chou and Lee (2016)	**√**			**√**
D'Amico and Lalonde (2017)		**√**		**√**
Ziadat (2022)			**√**	**√**
Durrani (2021)			**√**	
Durrani (2020)			**√**	
Holt et al. (2024)	**√**		**√**	
Huili et al. (2023)	**√**	**√**	**√**	
İnci and Saǧlam (2025)	**√**			**√**
Koo and Thomas (2019)	**√**		**√**	
Lee et al. (2024)	**√**			**√**
Lipska and Leś (2023)		**√**	**√**	**√**
Lith (2017)		**√**	**√**	**√**
Schweizer et al. (2019)		**√**	**√**	**√**
Schweizer et al. (2020)		**√**	**√**	**√**
Schweizer et al. (2022)		**√**	**√**	**√**
Schweizer et al. (2017)		**√**	**√**	**√**
Thayer and Bloomfield (2021)			**√**	**√**

Each row represents one included study. Some studies contributed to more than one thematic domain; therefore, the thematic categories are not mutually exclusive.

Table 4 lists the journal outlets in which the included studies were published. Many publications appeared in specialized art therapy and autism-related journals, such as Art Therapy, The Arts in Psychotherapy, and Advances in Autism. Several studies were also published in broader clinical and pediatric journals, including Clinical Case Studies, Frontiers in Psychiatry, and Clinical and Experimental Pediatrics. In addition, some publications appeared in regional or multilingual journals, such as Aktualnosci Neurologiczne, Paediatrica Indonesiana, and the Australian Occupational Therapy Journal. The journal distribution spans specialized, clinical, and regional academic venues.

**Table 4:** Journal title.

Journal	2016	2017	2019	2020	2022	2023	2024	2025
Advances in Autism						1		1
Aktualności Neurologiczne						1		
Art Therapy		2	2	1				
Australian Occupational Therapy Journal							1	
Children					2			
Children and Youth Services Review				1				
Clinical and Experimental Pediatrics						1		
Clinical Case Studies				1				
Education and Training in Autism and Developmental Disabilities	1							
Frontiers in Psychiatry						1		
International Journal of Art Therapy			1					1
International Journal of Instruction					1			
Paediatrica Indonesiana							1	
PLOS ONE							1	
The Arts in Psychotherapy				1				

### 3.2 Themes

This section reports the thematic findings derived from the included studies. Themes were identified through iterative coding and narrative synthesis of recurring intervention components and outcome domains across studies. Four themes were identified: (1) Emotional Expression and Regulation, (2) Social Skills Development, (3) Creative and Nonverbal Intervention, and (4) Sensory and Relational Integration (Figure 4). These themes represent recurring domains within the literature, and several studies addressed more than one domain. These thematic findings address the research questions concerning methodological characteristics and how art therapy interventions are associated with developmental outcomes in children with ASD.

**Figure 4:**
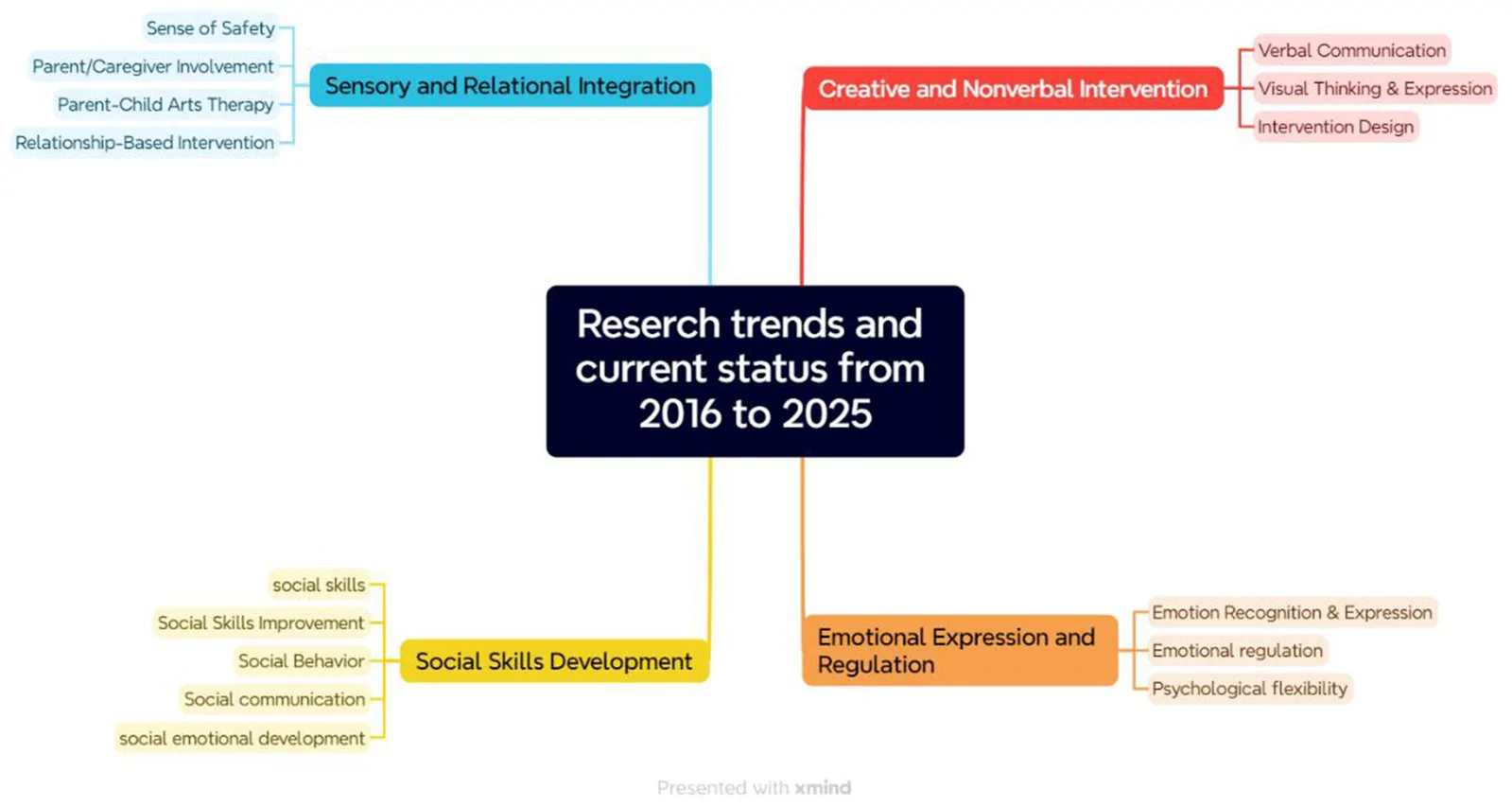
Overall network.

#### 3.2.1 Emotional expression and regulation

Under the theme of Emotional Expression and Regulation, the included studies describe art therapy as a context in which children with ASD engage in visual and multisensory forms of emotional expression. Compared with language-based interventions, visual art creation was presented as an alternative channel aligned with the visual processing characteristics of children with ASD, potentially offering opportunities for emotional expression beyond verbal communication (Lith, 2017). Huili et al. (2023) reported that visual stimuli featuring cool tones, high brightness, and high saturation appeared to capture and sustain the attention of children with ASD. Positive emotional responses were noted in kinesthetic interactive games incorporating immediate feedback and reward mechanisms, with observations involving emotional regulation and social interaction. Similarly, picture books with clear structures, concise language, and sequential narrative visuals corresponded with visual attention, communication, and imaginative abilities (Huili et al., 2023).

Regarding intervention outcomes, several intervention-based and follow-up studies reported changes in emotional regulation after participation in art therapy programs. Schweizer's Images of Self project reported that children showed substantial and moderate positive behavioral shifts in emotional regulation dimensions after completing treatment, with similar patterns observed over time (Schweizer et al., 2020). In several qualitative reports, parents, teachers, and therapists described children as appearing “happier, more stable, and better able to verbalize their experiences”, with corresponding increases in self-esteem and self-efficacy (Schweizer et al., 2019, 2022). In a case-based intervention study, collaborative tasks such as pulp modeling provided opportunities for expressing personal ideas and practicing turn-taking, sharing, and listening, alongside reductions in emotional outbursts (D'Amico and Lalonde, 2017). Lipska and Leś (2023) similarly suggested that art therapy was linked to changes in coping with anger and anxiety, greater behavioral flexibility, and adaptations in learning skills.

Visually and sensorily tuned art-making, combined with structured feedback and caregiver-supported practice, was discussed for emotional awareness and self-regulation among children with ASD, with some studies reporting similar observations beyond the therapy setting.

#### 3.2.2 Social skills development

Building on the emotional processes described above, the following theme focuses on the development of social skills in art therapy contexts. Changes in social interaction and communication behaviors were reported in children with ASD following art therapy interventions, with additional developments in social and emotional skills reported in studies using Developmental, Individual-differences, Relationship-based (DIR)–oriented approaches (Thayer and Bloomfield, 2021). Two experimental studies by Abdulah reported changes in social communication and reductions in restricted and repetitive behaviors following short-term intensive group art interventions (Abdulah et al., 2023). Similarly, online and offline art activities were linked to joint attention and social interaction in several reports (Ziadat, 2022).

In the Images of Self series, longitudinal and evaluation-based studies reported shifts in children's social behavior and flexibility. Qualitative reports further described children as communicating more frequently, seeking help more actively, and demonstrating greater self-confidence (Schweizer et al., 2020). Repeated case studies linked collaborative art tasks with task-oriented cooperation, help-seeking, and joint attention, alongside positive changes in social behavior and flexibility (Schweizer et al., 2022). Therapists' experiences also described changes in social communication behaviors, flexibility, and self-image (Schweizer et al., 2017).

Changes in children's participation, self-assertion in social interactions, communication, and cooperation skills were observed alongside reductions in hyperactivity and inattention following art therapy (D'Amico and Lalonde, 2017). A quasi-experimental study of İnci and Saǧlam's (2025) interactive art activity program reported positive quantitative findings related to social skill development. In a small-scale study involving two children, changes were reported across all three targeted social behaviors following a behaviorally oriented art program. The Digital Art Mentor Program created peer interaction spaces aligned with adolescents' interests through collaborative creation and daily socialization activities (Chou and Lee, 2016). Lee's Digital Art Mentorship Program further extended socialization activities to include snack time and collaborative creation, with interest-driven peer interactions corresponding with social development among adolescents with autism (Lee et al., 2024).

Shifts in communication, cooperation, joint attention, social confidence, and behavioral flexibility were observed in children with ASD across both therapeutic settings and everyday contexts such as home and school.

#### 3.2.3 Creative and nonverbal intervention

In addition to emotional and social dimensions, art therapy also involves creative and nonverbal processes. Art therapy, centered on visual and nonverbal creative media, as mentioned earlier regarding the visual-first cognitive processing characteristics of children with ASD (Koo and Thomas, 2019), was described as offering opportunities for expression and communication through visual and nonverbal forms (Chapman and Evans, 2020). Design elements such as kinesthetic interactive games, cartoon-style flat visuals, and simplified operational icons were described as capturing attention and commonly appearing in activities involving participation among children with ASD (Huili et al., 2023). The creative process involved the manipulation of diverse 2D and 3D media, a practice associated with motor skill development (Koo and Thomas, 2019), while studies have also identified potential links to sensory engagement and self-regulatory experiences (İnci and Saǧlam, 2025).

Qualitative and case-based studies linked creative activities using metaphors, imagery, and storytelling to nonverbal forms of communication. These forms of communication rely less on language and provide alternative channels for expressing emotions and needs through non-verbal means, which coincided with peer interaction and social participation in several studies. Furthermore, some studies noted reductions in social anxiety during nonverbal and creative activities (Chapman and Evans, 2020). As a mixed-methods study protocol, Holt et al. (2024) proposed a STEAM (Science Technology Engineering Art Mathematic) art-narrative workshop designed to examine creative self-efficacy, psychological empowerment, and design thinking among autistic youth. Inci's interactive art project identified quantitative changes in creativity expression and social skills, alongside reductions in repetitive behaviors (İnci and Saǧlam, 2025).

Art therapy interventions were described as involving multisensory engagement, visual metaphors, and creative narratives. Reported findings included sensory-motor integration, abstract thinking, and expanded nonverbal expression.

#### 3.2.4 Sensory and relational integration

Another important aspect identified in the literature is the relationship between sensory regulation and interpersonal engagement in art therapy contexts. Qualitative and relationally oriented studies described the use of multisensory art activities and mirroring interactions, in which therapists reflect children's actions, emotions, or expressions during creative activities. The use of art materials with varying colors, textures, and tactile qualities was described as helping children respond to different sensory experiences, with observations related to changes in sensory responsiveness (Durrani, 2020). Existing studies reported that actions such as tearing, cutting, drawing, and touching in tactile interactive games and structured art sessions were accompanied by observations involving hand-eye coordination, fine motor activity, and sensory integration, with some reports noting reduced hypersensitivity to unfamiliar stimuli (Koo and Thomas, 2019).

The artistic process frequently involved shared attention and emotional attunement within therapist–child interactions. Therapists may mirror the child's body language and artistic expression during nonverbal engagement and relational interaction. Some studies described these interactions as resembling attachment-related patterns of responsive communication (Schweizer et al., 2019). DIR-oriented creative art therapy emphasized tailoring materials and pacing to each child's sensory-emotional profile within ongoing therapeutic interactions (Thayer and Bloomfield, 2021). In a qualitative study of parent-child art therapy, these processes were extended to the family context. Collaborative creation allows parents and children to express unconscious emotions and memories through multisensory experiences involving touch, sight, and smell, with parents reporting increased reflection, empathy, and interaction during collaborative activities regarding their child's inner experiences (Bitan and Regev, 2022).

Several studies described art therapy as involving multisensory experiences, shared attention, and responsive interpersonal interaction. These processes coincided with observations involving social engagement, emotional expression, and self-regulation in children with ASD.

### 3.3 Creative–relational art therapy framework (CRATF)

The CRATF is proposed as a conceptual framework derived from the reviewed literature and should be empirically tested in future studies (see Figure 5). It integrates four themes: emotional expression and regulation, social skills development, creative and nonverbal intervention, and sensory–relational integration. These themes were synthesized into a conceptual structure encompassing emotional, social, creative, and sensory dimensions.

**Figure 5:**
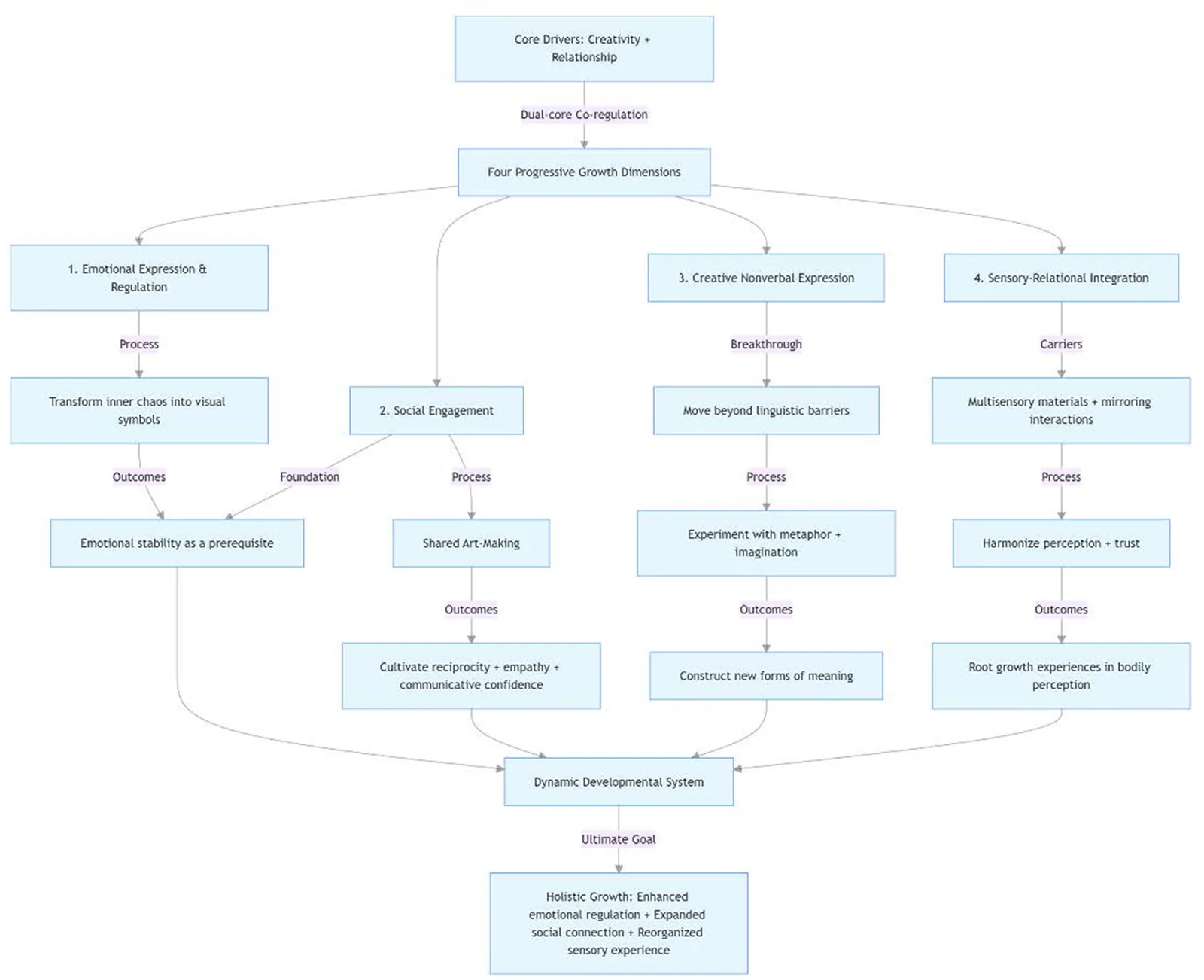
The conceptual framework of creative–relational art therapy framework (CRATF).

At the level of emotional expression and regulation, the reviewed studies described art-making as a context in which children externalized internal experiences and engaged in emotional expression and self-regulation. Emotional expression and regulation frequently occurred alongside social participation within shared art-making contexts characterized by reciprocity, empathy, and communicative confidence. Creative and nonverbal forms of expression were associated with visual, metaphorical, and narrative modes of expression that involved limited verbal communication demands. The sensory-relational dimension incorporated multisensory materials and mirroring interactions, and frequently appeared alongside descriptions of perceptual regulation and relational trust within the therapist–child–image dynamic.

The CRATF reflects recurring patterns identified in the reviewed studies, in which emotional regulation, social engagement, creative expression, and sensory-relational processes co-occur. It should therefore be understood as a conceptual framework rather than an empirically validated model.

## 4 Discussion

### 4.1 Purpose and the main findings of the study

This systematic review examined empirical studies on art therapy for children with ASD published between 2016 and 2025. Employing the PRISMA-guided screening, 22 studies meeting the CCAT quality criteria were included. The findings indicate a gradual increase in publication volume over the past decade, with contributions from Europe, North America, and Asia. This pattern suggests a growing international research interest in art-based interventions for ASD, although the overall number of empirical studies remains limited. The geographic distribution also reflects the concentration of research activity in regions with established clinical and educational research infrastructures.

Thematic analysis identified four themes: Emotional Expression and Regulation, Social Skills Development, Creative and Nonverbal Intervention, and Sensory and Relational Integration. These interrelated themes were synthesized into the CRATF, which organizes emotional regulation, social engagement, creative expression, and sensory–relational processes within a synthesized conceptual framework. Across the reviewed studies, these dimensions were linked with self-regulation, communication, and adaptive behaviors in children with ASD. The CRATF provides a synthesized interpretation of how emotional, social, creative, and sensory–relational dimensions may interact within art therapy practice.

Findings across the reviewed studies suggest that art therapy, through visual, multisensory, and relationship-based activities, may be related to emotional recognition, expression, and regulation in children with ASD. Reported findings also include changes in joint attention, cooperation, and social confidence, alongside differences in stereotyped and repetitive behaviors. Several studies described changes in sensory integration and parent-child interaction patterns following art-based interventions. While some studies reported similar patterns across home and school contexts, the extent of generalization remains variable.

Recent research has explored innovative approaches, including kinesthetic interactive games, parent-child co-creation, DIR-oriented interventions, and STEAM art-narrative workshops. These approaches reflect diversification in intervention formats, with increasing incorporation of interactive and digital elements. Further empirical investigation is required to determine their longer-term applicability across contexts, particularly given the relatively small and heterogeneous evidence base identified in this review.

### 4.2 Theoretical contributions

This systematic review contributes to the theoretical discussion of art therapy for children with ASD by organizing empirical findings into a structured framework. By identifying and synthesizing four core themes, namely Emotional Expression and Regulation, Social Skills Development, Creative and Nonverbal Intervention, and Sensory and Relational Integration, the review illustrates how these dimensions may be conceptually related within art therapy practice. Rather than treating these domains as separate outcomes, the framework presents them as interrelated aspects of intervention practice.

Second, the review extends existing perspectives by explicitly incorporating sensory and relational elements into the conceptualization of art therapy. The emphasis on sensory regulation and relational engagement as integral components of art therapy interventions contributes to a broader understanding of the therapeutic process. This perspective suggests that art therapy interventions involve attention to sensory processing and relational dynamics alongside emotional and social components. However, the current evidence base remains relatively limited and heterogeneous.

Third, the review contributes to the theoretical discourse by proposing a new conceptual framework for art therapy interventions. This framework synthesizes findings suggesting that art therapy may occur in multisensory, relationship-oriented therapeutic environments. Within such contexts, children engage in creative expression while participating in synchronous interactions and emotional attunement. Existing studies have reported social and emotional skills within art therapy interventions. This framework may offer a conceptual reference for future research and intervention design.

### 4.3 Practical implications

The findings of this review provide several considerations for the implementation of art therapy interventions for children with ASD. One practical consideration is the incorporation of diverse sensory materials and activities into their therapeutic sessions. Materials with different textures, colors, and tactile qualities may assist children with ASD in responding to sensory input. Interactive games and activities that involve hand-eye coordination and fine motor skills may be linked with sensory integration and motor development.

Another consideration is that art therapy interventions may encourage relational engagement and emotional attunement. Therapists can create opportunities for synchronous interactions and mirror children's artistic expressions as part of synchronous and relational interaction processes commonly discussed in relation to social skill development in children with ASD. Involving parents and caregivers in the therapeutic process may extend intervention experiences to home environments and encourage continuity across contexts.

A further implication relates to the heterogeneity of ASD. A comprehensive assessment of sensory preferences, cognitive abilities, and individual interests can inform the adaptation of intervention strategies. Tailored approaches may increase engagement and relevance for individual children.

An additional area involves integrating digital technologies into art therapy practice. Virtual reality (VR) and artificial intelligence (AI) may be used to support interactive art experiences within structured therapeutic settings. These technologies can complement traditional art therapy techniques when applied thoughtfully in relation to therapeutic goals. For example, VR may be utilized to design controlled sensory environments, while AI may provide adaptive or real-time feedback during art-making activities. Further research is needed to evaluate their clinical applicability and long-term impact.

### 4.4 Limitations and future studies

This review has several limitations that should be considered when interpreting its findings. The search strategy was limited to three major databases and English-language publications, which may have excluded relevant gray literature, book chapters, conference papers, and non-English studies. As a result, community-based art therapy programs and digital interventions in non-English-speaking regions may be underrepresented. Expanding database coverage and including multilingual sources would enhance the comprehensiveness of future reviews.

Publication bias is also acknowledged. Studies with small samples or non-significant outcomes may be less likely to appear in peer-reviewed journals, potentially shaping the overall evidence profile. Future reviews may address this by incorporating unpublished studies, preprints, and meta-analytic tools designed to examine publication bias.

Besides, although this review covers literature from 2016 to 2025, newly published studies may not yet be fully indexed. This may limit visibility of emerging trends such as somatic interaction, VR art-making, and AI-supported creative tools. Ongoing updates of systematic reviews are therefore important to capture recently indexed research.

Building on these limitations, future research can advance in several key directions. Longitudinal studies may provide further insight into developmental trajectories beyond immediate post-intervention outcomes, as suggested by D'Amico and Lalonde's (2017) findings on long-term improvements in self-regulation and participation. Digital innovation represents another important direction, with VR and AI introducing structured and interactive formats that may benefit from systematic evaluation (Holt et al., 2024; Huili et al., 2023). Cross-cultural research remains critical, given that cultural context may influence the implementation and interpretation of interventions, as illustrated by Koo's analysis of culturally embedded nonverbal expression (Koo and Thomas, 2019). Comparative studies examining traditional and technology-assisted art therapy formats may provide further insight into differential outcomes. Parent–child co-creation models also merit continued investigation in light of evidence linking collaborative art processes to relational attunement and emotional communication (Bitan and Regev, 2022).

Future research may contribute to a more refined understanding of intervention practices and contextual variation in art therapy.

## 5 Conclusion

This systematic review synthesized 22 empirical studies on art therapy for children with ASD published between 2016 and 2025. Across the reviewed literature, four recurring themes were identified: (1) emotional expression and regulation, (2) social skills development, (3) creative and nonverbal intervention, and (4) sensory–relational integration. The themes reflect the multidimensional nature of art therapy practice within clinical and educational contexts.

The review further identifies key intervention processes, including multisensory input tuning, nonverbal externalization, relational synchrony, motivational engagement, and structured social practice. These processes provide a conceptual perspective on how art therapy interventions are associated with emotional, social, creative, and sensory processes. The findings suggest that art therapy programs may benefit from incorporating children's sensory preferences and creative motivations, integrating structured feedback and caregiver involvement, and exploring digital or gamified modalities to support engagement.

This study proposes the CRATF as a preliminary conceptual framework that integrates emotional, social, creative, and sensory–relational dimensions of art therapy practice. Future longitudinal, experimental, and mixed-methods studies are needed to empirically test, refine, and validate this framework.

## Data Availability

The original contributions presented in the study are included in the article/Supplementary material, further inquiries can be directed to the corresponding author.
